# Patient-centeredness in the multimorbid elderly: a focus group study

**DOI:** 10.1186/s12877-021-02448-8

**Published:** 2021-10-18

**Authors:** Manuela Kanat, Jonas Schaefer, Laura Kivelitz, Jörg Dirmaier, Sebastian Voigt-Radloff, Bernhard Heimbach, Manuela Glattacker

**Affiliations:** 1 Section of Health Care Research and Rehabilitation Research, Institute of Medical Biometry and Statistics, Faculty of Medicine and Medical Center - University of Freiburg, Hugstetter Str. 49, 79106 Freiburg, Germany; 2 Department of Medical Psychology, University Medical Center Hamburg-Eppendorf, Martinistraße 52, 20246 Hamburg, Germany; 3 Center for Geriatric Medicine and Gerontology Freiburg, Institute for Evidence in Medicine (for Cochrane Germany Foundation), Faculty of Medicine and Medical Center - University of Freiburg, Breisacher Str. 86, 79110 Freiburg, Germany; 4 Center for Geriatric Medicine and Gerontology Freiburg, Faculty of Medicine and Medical Center - University of Freiburg, Lehener Str. 88, 79106 Freiburg, Germany

**Keywords:** Patient-centeredness, Focus group, Older patients, Multimorbidity, Chronic disease

## Abstract

**Background:**

Patient-centeredness (PC) aims to adapt health care to the individual needs and preferences of patients. An existing integrative model of PC comprises several dimensions of PC which have not yet been investigated from the patients’ perspective. Older patients with multimorbidity represent a target group for patient-centered care, as their care needs are particularly complex and should be addressed individually. We aimed to assess the perspective that older patients with multimorbidity have of patient-centered care and to examine the transferability of the integrative model of PC to this specific population.

**Method:**

We performed 4 guided focus group interviews with a total of 20 older individuals with multimorbidity. The focus group interviews were audio-recorded and transcribed verbatim. Patients’ statements were content-analyzed applying an a priori designed system of categories that included the dimensions of PC from the integrative model and the additional category ‘prognosis and life expectancy’, which had emerged from an initial literature search on aspects of PC specific to the multimorbid elderly.

**Results:**

The new category ‘prognosis and life expectancy’ was confirmed and expanded to ‘individual care needs related to aging and chronic disesase’. All dimensions of our integrative PC model were confirmed for older patients with multimorbidity. Among these, we found that eight dimensions (individual care needs related to aging and chronic disease, biopsychosocial perspective, clinician-patient communication, essential characteristics of the clinician, clinician-patient-relationship, involvement of family and friends, coordination and continuity of care, access to care) were complemented by aspects specific to this target population.

**Conclusions:**

The integrative PC model is applicable to the population of older patients with multimorbidity. For a population-specific adaptation, it might be complemented by the dimension ‘individual care needs in aging and chronic disease’, in conjunction with age-specific aspects within existing dimensions. Together with corresponding results from a Delphi survey, our adapted PC model will serve as the basis for a subsequent systematic review of instruments measuring PC in older patients with multimorbidity.

**Trial registration:**

PROSPERO (https://www.crd.york.ac.uk/prospero; CRD42018084057; 2018/02/01), German Clinical Trials Register (www.drks.de; DRKS00013309; 2018/01/23).

**Supplementary Information:**

The online version contains supplementary material available at 10.1186/s12877-021-02448-8.

## Background

Patient-centeredness has been defined as a crucial prerequisite for high-quality health care [1]. It refers to the individualization of health care processes by considering the specific needs and preferences of the individual patient and supporting their active participation in health care decisions taking into account the patients’ preferences [2, 3]. Recently, there has been a shift in terminology from ‘patient-centeredness’ to ‘person-centeredness’ [2, 4]. However, while the two concepts are similar, they differ regarding the receiver as well as the goal of care: Patient-centeredness (PC) targets sick persons and disease-treatment processes and aims at ensuring a functional life for the patient. In contrast, person-centeredness may also imply healthier individuals in the context of health promotion and aims at creating a meaningful life for persons [5, 6].

Within the past years, substantial efforts have been made to implement PC at different levels of health care [7]: patient rights have been strengthened by legislation (macro level), institutions providing patients with evidence-based health information and aids for shared-decision making have been established (meso level), and interventions promoting PC within patient-physician-interactions have been implemented (micro level) [7, 8]. Preliminary evidence suggests that patient-centered care may reduce healthcare utilization and medical care costs [9, 10], and improve health behavior as well as patients’ satisfaction [8, 11].

Multimorbidity, as defined by the co-existence of two or more chronic conditions, is associated with increased mortality, resource utilization, and higher health care costs [12–14]. Elderly patients with multimorbidity have been identified as a primary target group for patient-centered care approaches, as current treatment guidelines are mostly disease-specific and are supposed to be inappropriate for addressing the complex health care needs in patients with multiple chronic diseases [15–19]. Due to the demographic trend of aging populations, the number of elderly patients (aged 65 years and older) will likely rise in the future [20], with over half of them suffering from multimorbidity [19, 21–23]. Notably, the health care needs and preferences of older patients with multimorbidity tend to differ greatly from those of younger patients. For example, while the majority of older patients with multimorbidity still prefers active involvement in care decisions, the overall preference for a participatory communication style decreases, both with age, and the number of medical conditions [24–28]. In this context, adapting the healthcare system to the specific characteristics and needs of older patients with multimorbidity has been declared a major imperative [29, 30].

The American Geriatrics Society has established guiding principles for the patient-centered care of older adults with multimorbidity [19]. These include eliciting patient preferences and carefully weighing up individual burdens, risks, benefits, and prognosis. Similarly, guidelines on multimorbidity proposed by the German College of General Practitioners (GP) and Family Physicians (DEGAM) include the valuing of specific health care goals related to multimorbidity such as minimizing the side-effects of medication, or maintaining social activities [31]. Overall, current guidelines on multimorbidity and polypharmacy include recommendations that reflect central aspects of PC such as the incorporation of patient preferences or individualized treatment management (for a current review, see ([32])).

However, developing and evaluating patient-centered interventions for older patients with multimorbidity may be corrupted by inconsistencies in how the concept PC is defined and operationalized [2]. For example, Robinson and colleagues [3] pinned PC down to two fundamental characteristics (patient involvement in care, and the individualization of patient care) whereas Mead and Bower [33] defined PC by five conceptual dimensions (biopsychosocial perspective, patient-as-person, sharing power and responsibility, therapeutic alliance, and doctor-as-person). A concept analysis by Morgan and Yoder [34] revealed four defining attributes (holistic, individualized, respectful, and empowering). Our research group has proposed one of the most comprehensive PC models [35]. We reviewed existing definitions on PC in the literature and integrated their results within our ‘integrative model of PC’ [35] comprising 15 dimensions, which reflect principles, enablers, and activities of PC at the micro and meso level of health care (e.g., relationship between patient and care provider, access to care, coordination and continuity of care). Our model was recently evaluated from the patients’ perspective (patients having at least one chronic disease (e.g., cancer or cardiovascular disease)) who considered each of the model’s dimensions as relevant [36].

However, the transferability of our integrative PC model to the specific population of older patients with multimorbidity has not been investigated yet. In this context, it remains unclear whether our literature-based model concurs with our target group's perspective on core elements of PC. The purpose of this focus group study was therefore to explore the concept of PC from the perspective of older patients with multimorbidity. We intended to reveal aspects of PC which are particularly relevant for this group but may not be covered by the existing integrative PC model. This may serve as the basis for adapting our model to the specific healthcare needs and preferences of a highly relevant patient population.

## Methods

This study is part of a larger project in which patients’ and experts’ considerations of PC in older patients with multimorbidity were assessed in two different studies. Results from both will be used to adapt the integrative PC model [35] to the needs of this specific patient population. The adapted model will in turn serve as the basis for a subsequent systematic review of instruments to assess PC in this population [37]. The present article focuses on the first part of the project, namely, the perspective of older patients with multimorbidity on patient-centered care. Reporting follows the Consolidated criteria for reporting qualitative research [38] (see supplementary COREQ checklist, Additional File 2).

### Participants

We recruited patients via the Center for Geriatric Medicine and Gerontology (ZGGF) at the University of Freiburg, which offers outpatient service for frail and non-frail patients with age-associated disorders (e.g., decrease in memory capacity, gait disturbances). Eligibility for participation was fulfilled if patients were aged 65 years or older and suffered from multimorbidity (as defined by at least two chronic medical conditions). To allow for group heterogeneity regarding sex, age, and diagnoses, we applied a purposeful sampling method. Exclusion criteria for participation were any severe cognitive impairment (as diagnosed by trained staff at the ZGGF beforehand) and insufficient language skills. Suitable patients were contacted personally during treatment in the clinic or by telephone and informed via information flyers. While we have no information on how many patients were contacted overall, the purposeful sampling was not stopped until an over-recruitment was achieved to ensure that we obtained a sample size of at least 20 patients. We ultimately recruited 26 patients. Of those 26, 3 patients had dropped out their participation before the focus group date, whereas the other 3 failed to show up on the date of the interview with no explanation. Our final sample consisted of 20 patients (13 male, 7 female) aged between 69 and 90 years (see Table 1). An overview of the sample’s diagnoses according to ICD-10 is illustrated in Additional File 1. On a rating scale ranging from 0 = ‘no burden at all’ to 7 = ‘very heavy burden’, 70% of the patients rated their subjective burden of disease as ‘heavy’, ‘rather heavy’ or ‘very heavy’ (M = 4.8, SD = 1.4).

**Table 1:** Sample characteristics (*n* = 20)

Characteristic	
Age, mean (SD)	76.8 (5.1)
Female, n (%)	7 (35%)
Married or living with significant other, n (%)	16 (80%)
Employment, n (%)	
Employed	3 (15%)
Retired	17 (85%)
Education, n (%)	
Qualification for university entrance *[Hochschulreife]*	5 (25%)
Advanced technical college entrance qualification *[Fachhochschulreife]*	4 (20%)
General certificate of secondary education *[Mittlere Reife]*	4 (20%)
Secondary general school certificate *[Hauptschulabschluss]*	7 (35%)
Duration of primary illness, n (%)	
Less than 5 years	5 (25%)
5 to 10 years	5 (25%)
More than 10 years	6 (30%)

*SD* standard deviation

### Data collection

In June 2018, we performed 4 focus group interviews, each containing 3 to 8 patients. The interviews took place at the ZGGF and were digitally audio-recorded. Patients were given the moderator’s name and professional background and received oral and written information on the research interest related to the study as well as the procedure of the focus group discussion. Written informed consent was obtained from all patients before the focus group started. Patients were interviewed by one of two researchers: JS is a male researcher who holds a master’s degree in health and nursing science and has worked with older patients during his former profession as a nurse on thoracic surgery wards. MK is a trained, female psychologist and a postdoctorate researcher. During and after the interview, hand-written field notes were made on emerging topics and pecularities. After the interview, the patients answered a short questionnaire on socio-demographic and illness-related characteristics (Additional File 3). The questionnaire was developed for this study and has not been published elsewhere. In all, each session lasted about 2 h with the interview accounting for approximately 1 h.

### Interview guide

The semi-structured interview was based on an interview guide (Additional File 4) that our research team had developed beforehand according to recommendations by Krueger and Casey [39]. The interview was initiated with an open question on patients’ concept of the term PC to assess personally relevant aspects of PC while avoiding a bias by preset specifications (see Table 2). The following questions targeted aspects of PC that might be specifically relevant to the population of older patients with multimorbidity and their relatives’ views. In addition, we included optional deeper questions on specific dimensions of the integrative PC model as well a final question asking for any uncovered topic relevant to PC.

**Table 2:** Focus group questions

Targeted aspect of patient-centeredness	Central question
Lay concept	*What spontaneously comes to mind when you think of the term patient-centeredness?*
Specifities of older patients	*If you think of patient-centered health care, what might be different for older as compared to younger patients?*
Specifities arising from multimorbidity	*From your perspective, what should be considered in the health care of older patients who suffer from multiple chronic diseases?*
View of relatives	*What might your relatives say about what needs to be specifically considered in the health care of older patients who suffer from multiple chronic diseases?*

### Data analysis

Once a member of the research team had anonymized any person-related information by deleting it from the audio files, files were sent to an independent agency for transcription. The transcripts were analyzed independently by two researchers (MK and JS) to avoid subjective biases during coding. The analysis was conducted in ATLAS.ti (https://atlasti.com/) and followed the principles of Mayring’s structuring content analysis [40]. Within the coding system, categories were predefined according to the 15 dimensions of PC as proposed by the integrative model of PC [35]. Preceding our focus group study, we had conducted an explorative literature search on further PC aspects that might be particularly relevant to our target group. This search revealed ‘prognosis, life expectancy, and treatment burden’ as additional aspects of PC for our target group. These were validated within a Delphi-survey with experts in geriatric health care (in round 1 with *n* = 48 experts, in round 2 with *n* = 39 experts) [41] and included as a preliminary category within our coding system. Coding rules were defined to resolve conflicts in case of reduced identifiabilty of categories. These rules were refined during the analysis in an iterative manner and were used to allocate text passages to the predefined categories. The content analysis entailed several steps. In the first, all transcripts were screened for relevant text passages. Those text passages were paraphrased and then generalized according to the summarizing content analysis by Mayring [40]. In the next step, the generalized text passages were coded. After the corporate revision of these codes and refining the coding rules, the coding system was finalized and codes assigned to the predefined categories representing different dimensions of PC (structuring content analysis). In addition to this deductive procedure, we planned to take an inductive approach in case a text passage failed to match any of the given categories. Our final coding system comprised the definition of each category together with an exemplary participant comment. Discrepancies in the assignment of coders were discussed by these two researchers and resolved by direct consensus or consultation with a third researcher (MG).

## Results

All topics emerging from the focus group interviews could be assigned to one of our predefined categories, including the new category ‘prognosis, life expectancy, and treatment burden’ (see Table 3). While coding, further aspects were added to this category, and we renamed it ‘individual care needs related to aging and chronic disease’ within the final coding system. No other categories came up taking an inductive approach. The PC dimensions address most extensively the relationship between patient and clinician, access to care, as well as coordination and continuity of care, and patient information. In contrast, patients seldom refer to specific communication techniques of clinicians, or aspects regarding teamwork and teambuilding. The PC aspects emphasized most or specifically related to age and multimorbidity by our patients are presented in detail and exemplifed through our participants’ quotes. Owing to space constraints, the remaining topics are depicted in Table 3 only.

**Table 3:** Topics derived from the focus groups and assignment to categories (i.e. dimensions of patient-centeredness) and levels of health care

**Micro level of care** **(patient interaction**)		
**Category**	**Description**^a^	**Associated topic during discussion**
Patient as a unique person	*Recognition of each patient’s uniqueness (individual needs, preferences, values, feelings, beliefs, concerns and ideas, and expectations).*	Individual patient characteristics (e.g., health status, personality) Individual health care preferences (e.g., related to specific treatment, medication) Usefulness of patient decrees Individualization of health care / treatment plans
Individual care needs related to aging and chronic disease	*Refers to the individual prognosis, life expectancy, treatment and disease burden, as well as natural aging processes.*	Prognosis Individual treatment / disease burden Age-specific anxiety / uncertainty Aging process / gradual frailty / dependence Polymedication: check for interaction between drugs More time needed for health care visits of older multimorbid patients Preference for ‘in-home nursing’, e.g., by nonprofessional, foreign nurses (including structural and financial preconditions)
Biopsychosocial perspective	*Focuses on the development and context of an individual patient,* i.e. *non-medical and relatively stable characteristics of a patient and his living situation.*	Living situation (e.g., home nursing, sick relative) Availability of and support by relatives Loss of partner / loneliness
Clinician-patient communication	*Refers to concrete or even observable aspects of communication that may, in turn, shape the relationship between patient and clinician.*	Personal experiences of failed / poor communication with clinician (e.g., clinician vehemently tries to alter the patient’s decision) Clinician: ask the patient questions (e.g., about side-effects of medication) Clinician: encourage the patient to utter doubts or contradictory views Clinician: include a personal instead of a mere professional communication style
Essential characteristics of the clinician	*Comprises personal characteristics of the clinician that patients describe as desirable.*	Empathy, commitment, forthrightness Expertise, specialization, experience
Clinician-patient-relationship	*Refers to a central principle of patient-centeredness, namely a good relation and professional partnership between patient and clinican.*	Clinician: Patience and understanding Clinician: Respect for the patient (listen carefully, take patient seriously, care for patients’ needs) Trust between patient and clinician Clinician: Consider the patient’s individual life story
Patient involvement in care	*Considers the patient as an active collaborator of the clinician who should be actively involved in decisions on his health care as far as he prefers to.*	Preference for and positive experience with shared decision making Mismatch between the clinician’s communication style and the patient’s communicational preferences Clinician: Overstraining patients with increased responsibility and involvement
Patient information	*Describes the provision of patient-tailored health care information under considerance of the patient’s information needs and preferences.*	Visibility and comprehensibility of written information Information on treatment alternatives, side-effects of medication and prognosis Holistic approach: Information on disease effects on general condition, possible changes in behavior and consequences for everyday life Lists with contact persons / Uncertainty about whom to ask for information Information on waiting times at hospitals Consider informational preferences: Confusion and uncertainty by too few, too much or inadequate / contradictory information GPs as ‘translator’ of incomprehensible information
Involvement of family and friends	*Active involvement of and support for the patient’s relatives and friends to the degree that the patient prefers.*	Clinician: Provide relatives with information on the patient’s medical condition, implications of the disease for everday living Clinician: Ask relatives for information (e.g., about the patient’s medical preconditions)
Patient empowerment	*Recognition and active support of the patient’s ability and responsibility to self-manage his or her disease*	Clinician: Provide the patient with specific brochures and clinical recommendations on self-management options Clinician: Encourage patients to take over responsibility Clinician: Accept patient’s opinion Patient: Induce changes in lifestyle and health behavior, e.g., memory training, fitness course, healty diet Patient: Search for information, e.g., on health-related self-management activities Patient: ‘Look after oneself, especially in old age (e.g., avoid infections)
Physical and emotional support	*Recognition of the patient’s physical and emotional need and behaviors that address these needs*	Physical: Usefulness of home care services Physical: Availability, costs and ‘adaptability’ of technical advices such as walking frames, nursing beds Physical: Patient safety (intimate hygiene in hospitals) Inter- and intrapersonal variability of support needs Emotional (clinician): Responsiveness to patients’ fears Clinician: Recommend / prescribe additional or integrative therapies (e.g., psychotherapy) Clinician: Assess and account for support needs (e.g., physical impairments / disabilities of a patient) Emotional (clinician): Reduce the patient’s uncertainty by informing him
**Meso level of care** **(health care organization and community**)		
**Category**	Description^a^	**Associated topic during discussion**
Coordination and continuity of care	*Refers to the coordination between different care providers and services as well as the continuity of care (*e.g., *in terms of optimized transitions).*	GP as gate-keeper (coordinator, pooling of information) Importance of prearranging medical aftercare Importance of exchanging information between different care providers Usefulness of community health centers or joint practices due to integration of different professions Increasing responsibility of family members regarding the coordination of health care with progressing age and / or disease severity
Integration of medical and non-medical care	*Refers to the recognition and integration of non-medical aspects of care (*e.g., *patient support services) into health care services.*	Special prevention and after-care programmes Prescription / recommendation of non-medical care (e.g., homeopathy, psychotherapy, physiotherapy)
Teamwork and teambuilding	*Facilitation of effective teams characterized by a set of qualities (*e.g., *respect, trust, shared responsibilities, values, and visions)*	Insufficient communication between medical doctor and doctor’s receptionist
**Meso** **/** **macro level of care** **(health care organization and community** **/** **policy and regulations**)		
**Category**	Description^a^	**Associated topic during discussion**
Access to care	*Refers to the availability and timely accessibility of individually appropriate health care services and institutions (*e.g., *decentralized services).*	Patient: Insufficient knowledge about contact persons and institutions Institutions / policy: Impairments by bureaucracy Institutions: Waiting times for or at medical appointments, technical aids etc. Institutions: GP as first contact person in the health care system Institutions: Need for community health centers located in the neighborhood area Institutions / policy: Underpayment of health care staff Policy: Insufficient financing of medical services by insurers

*GP* general practitioner

^a^ Some of the descriptions have been adopted from Scholl et al. (2014)

Aspects of PC associated with the dimension ***individual care needs related to aging and chronic disease*** arise from multiple physical burdens and psychosocial stressors. Patients state problems associated with polymedication, e.g., that practitioners tend to prescribe multiple medications rather incautiously:

*„I’ve had to take eight to ten pills, and have lowered the number myself […]. My GP or other doctors, they don’t hesitate to pull out their prescription pad” (interview 3, line 153–156)*

Aging and multimorbidity are also associated with psychosocial distress, particularly the fear of a progressive loss of functions and growing dependency on others.

*„I worry about the dependency that can affect me, especially if I become mentally impaired. No longer being able to control what should happen is what I find worse.” (interview 1, line 335–337)*

One patient associates becoming dependent and needing care as “not feeling human anymore” (interview 1, line 82) indicating how strongly this state can affect the patient’s self-concept.

From a biopsychosocial perspective, patients find that the situational factor of living alone at old age and the absence of supporting partners promotes loneliness as well as depression, and that it raises the risk of (early) mortality.

*„The older I get, the harder I find being alone. […] I’m actually very well looked after. But still – you get home and find yourself all alone in your apartment. That sometimes feels like a blow. Now, the older I get, the more it gets to me.” (interview 2, line 675–680)*

Corresponding to this increased psychological vulnerability, patients appreciate ***essential characteristics of the clinician*** that facilitate personal rather than merely professional contact with patients (e.g., honesty, forthrightness) and prioritize aspects of the ***clinician-patient-relationship*** (e.g., trust, respect and cooperation) over clinicians’ expertise. However, patients feel that their specific communication needs are frequently not matched as many clinicians act under time pressure and are not interested in hearing older patients’ “rambling stories”.

*„Older people need more understanding. […] They need to be listened to. And they [the doctors] don’t feel like hearing your story or about your illnesses and life style.” (interview 4, line 286–289)*

Of note, some patients fear endangering their relationship with their clinician by speaking their mind and contradicting them. Thus, aspects of the relationship between clinician and patient have a direct impact on another dimension of PC, namely ***patient involvement****.* Some patients prefer to be actively involved in care processes and decisions (***patient involvement***).

*„…as a responsible adult patient. That we have ideas to contribute. It’s wrong to just accept everything they say. Rather, we have our own opinions and want to express them.” (interview 3, line 730–732)*

These patients positively refer to situations in which their clinician had actively asked for their consent, and they feel disrespected if the clinician ignores their opinion. In contrast, one patient expresses more critical thoughts about the increasing involvement of patients:

*„Well for one, there’s that buzz expression ‘responsible patient’, which I find most problematic, because I can get information through the internet or by talking with many doctors. But because I don’t have the background knowledge, I also get confused. Whether that is better than a patient who knows nothing and follows their doctor’s advice, I can’t say.” (interview 4, line 422–427)*

This comment implicates that ***patient information*** can also be confusing. Particularly regarding doctor’s reports, patients report insufficient understanding of the medical vocabulary which – in case of severe diagnoses – some found highly unsettling. Therefore, some patients expect their GP to translate incomprehensible medical information for them whereas others do not wish to be informed in detail, e.g., about a negative prognosis:

*„It might be quite all right if they don’t describe that in detail. What I don’t know might be that much better. If I know that I’ll be completely clueless in ten years, well, then, I’d rather not know.” (interview 1, line 239–242)*

Information on and knowledge about health care services and institutions on the other hand are generally appreciated. While the patients perceive their GPs as their first contact person and the most trusted care provider, they would like to have standardized information on other contact persons and health care institutions:

*„That I know whom I can turn to when I’m unwell and alone. When there’s no one at home or in my flat who will do that for me. […] that there’s address list available in every household.” (interview 1, line 711–717)*

Regarding the highly complex care needs of older patients with multimorbidity, patients highlight the importance of optimizing the ***continuity and coordination of care***. They consider regular medical follow-up visits with GPs and preventive medical check-ups to be highly important for chronically ill and older patients in order to keep track of changes in the course of a disease. Particular in case of polymedication, patients also expect their GPs to review the list of medications they take and to check for potential side-effects as well as risky interactions between prescribed drugs.

They describe situations in which the waiving of a careful examination (e.g., due to time pressure) may have dramatic consequences on older patients’ health status:

*„In any case, he didn’t ask what other [pills] I take. And then, right afterwards, I collapsed because the blood thinner I take caused a complication. […] and the emergency doctor had to come.” (interview 3, line 221–225)*

Moreover, patients regard a facilitated and regular exchange of information between different care providers to be an important aspect of PC for patients with multimorbidity. At the institutional level, they favor facilities that provide different caregivers and offer an integrated treatment program, e.g., community health or care centers.

*„It would be better if we had a care center in each neighborhood where many […] caregivers work during the day, who could be called and to whom I could say, could you please send somebody, I need this and that, can anybody help?” (interview 1, line 683–686)*

Patients state that such centers could optimize communication between professionals (e.g., via a common electronic health record) and break down barriers to accessing health care, as (older) patients would then not have to see different clinicians in other locations.

Besides official healthcare institutions, patients also consider their relatives to be important and indispensable pillars within health care of older patients as they keep track of relevant medical information, initiate contact with clinicians, or ensure they take their medications correctly. Patients therefore wish that family members were recognized as secondary caregivers within the health care process who should be informed and involved (***involvement of family and friends***). Beyond medical emergencies, they suggest informing relatives about the implications that their ill family member’s illnesses and medications may have for everyday life.

Overall, patients envision a shift in responsibility for their health care from themselves towards their relatives when they become more dependent or even bed-ridden. However, they associate this with organizational challenges along with the fear of becoming a burden to family caregivers.

*„It’d be difficult if we’re no longer independent – if we became bedridden, dependent. […] I don’t want to be stuck on the phone all day with my kids, can you come, can you put me to bed, can you do this or that…” (interview 1, line 653–662)*

Once they do need longterm care, patients generally prefer home nursing over assisted-living arrangements or nursing homes. However, they state several factors that might restrict accessibility to home nursing (***access to care***). For example, relatives may live far away or be too busy to nurse, or patients may not be able to afford the additional costs for outpatient nursing care services not covered by insurers or social welfare. Overall, patients see a demand for spending more public money to enable more at-home health care for older patients with multimorbidity so that they can maintain their self-management abilities and prevent even more costly institutional nursing care for as long as possible.

*„That saves money too. Because if I’m at home and looking after myself, that’s cheaper than if I were in a convalescent home or assisted living setup.” (interview 1, line 425–427)*

Lastly, patients complained that the access to requested health services such as home nursing services or technical support devices (e.g., a height-adjustable bed) are often slowed down by bureaucratic hurdles and long administrative channels. They advocate a more centralized structure with only one contact person (e.g., their GP) and one responsible institution (e.g., a central health care agency) in order to lower costs and accelerate the workflow.

## Discussion

The dimensions of the integrative PC model were confirmed for our target group of older patients with multimorbidity and further complemented by aspects specific to this target population. Hence, the integrative model of PC can be transferred to the care of older patients with multimorbidity. Population-specific aspects not covered by the existing model’s dimensions were assigned to the newly-proposed PC dimension ‘individual care needs related to aging and chronic disease’. This dimension was developed on the basis of an initial literature search and expert Delphi survey within our research project [41] and has now been confirmed from the patient’s perspective within the current study.

Overall, it became apparent that aging and living with multiple chronic health conditions induces heightened psychological vulnerability in patients (e.g., due to substantial disease burden or age-specific fears) which strongly influences their health care needs. Frailty, characterized by a decrease in multiple physiological functions and a heightened vulnerability towards stressors [42], was generally feared by many of our participants. In line with this finding, delaying frailty and promoting independence have recently been identified as key priorities of older patients with multimorbidity, carers, and health and social care providers [43]. In correspondence with this patient group’s heightened psychological needs, the quality of the relationship and of communication between patient and clinician was identified as one of the most crucial aspects of PC in elderly patients with multimorbidity. In line with results from a previous study, we found that this population hopes that providers will listen carefully to them, offer stable and supporting relationships, and acknowledge their needs [44].

However, previous studies have shown that these hopes are often not being adequately addressed. For example, the health needs as perceived by elderly patients often deviate strongly from the treatment priorities set by their clinicians [45, 46]. This reveals that the communication about patients’ individual needs and preferences and about incorporating them into a shared decision-making process on health care goals and decisions remains inadequate for older patients with multimorbidity. In line with this assumption, Wrede and colleagues [46] found that consultations with older patients with multimorbidity rarely reflect the assessment and shared determination of health priorities. On the other hand, it should be noted that patients’ individual preferences for autonomy and involvement vary widely [47]. In our study, some patients felt rather burdened by having to digest complex medical information and share responsibility with healthcare professionals. This is in line with evidence suggesting that older patients mostly prefer a patient-centered over a biomedical communication style, but are less likely to do so than younger patients [48].

Some older patients suffering from multimorbidity may tend to prefer a biomedical communication style because their personal values and expectations of clinical encounters differ from those of younger or less morbid patients. For example, they might prefer to rely on the physician as a medical authority to prevent themselves from suffering serious harm [49]. This seems likely, given the complexity of care demands and decisions and severity of some of these patients’ medical conditions. Against this background, aspects of a biomedical communication style may still be patient-centered, if they are willingly chosen after assessment of the patient’s individual communication preferences and goals of care. Based on such an assessment, implementing PC in the care of older patients with multimorbidity means building a bridge between respecting their potentially reduced preference for information-sharing and involvement, whilst still motivating and empowering them towards a minimum of active participation required to ensure optimal care of their chronic conditions [50]. Patient involvement in decision-making processes might be fostered by implementing patient-centered communication techniques, such as motivational interviewing and narrative counseling techniques [18].

Moreover, we found that older patients with multimorbidity desire a single provider who assumes responsibility for coordinating their care and for integrating care concepts to optimally address their complex care needs. Fleming and Haney [29] emphasize that such coordinated and integrated care should help to avoid the often fragmentary involvement of multiple institutions and physicians in the care of older patients.

Overall, the patient perspective on PC assessed in our study largely corresponded with recent recommendations from different expert groups and proposed care models on how to realize PC in older patients with multimorbidity, e.g., one primary point of contact within the healthcare team, active coordination and continual and integrated communication among all healthcare service providers, or supporting self-management in patients and families [2, 51].

To date, patient-centered care seems to correlate positively with physical and social well-being as well as satisfaction with care in patients with multimorbidity [52]. However, patient-centered interventions and comprehensive care models specifically adapted to and evaluated in individuals with multimorbidity remain scarce [51, 53]. Our study revealed several factors which might impede the implementation of patient-centered care or its accessability in the older, multimorbid population. Some of these barriers tend to be patient-related, such as reduced mobility or inadequate knowledge about services and contact persons, whereas others reflect the professional or organizational level, such as insufficient or inadequate funding, or the fragmentation of health services (compare 18, 50). Regarding the latter, Lau [54] further distinguished between operational barriers (e.g., limited time and resources), communication barriers (e.g., insufficient sharing of information) and perceptual barriers (e.g., perceived deficits in the work performance of other health care providers involved in care) within the care of the multimorbid elderly. Identifying and overcoming such barriers represents a major challenge when establishing patient-centered care for older patients with multimorbidity.

### Strengths and limitations

Previous Delphi surveys have validated the integrative model of PC from the perspective of experts [41, 55]. A recent study focussed on patients’ perceptions [36]. Our study is the first to compare its dimensions with patients’ perceptions of patient-centered health care in older patients with multimorbidity. Considering the rather deductive approach of our study and the fact that the emerging topics could be fully assigned to our pre-defined PC dimensions, data saturation may be assumed. Of note, our study findings suggest that the perceptions of multimorbid older people correspond closely with PC’s conceptual dimensions as experts have proposed [41], and with current guidelines for the patient-centered care of elderly people with multimorbidtiy [19]. Together, these different perspectives may be integrated within a population-specific adaptation of the PC model which will serve to develop patient-centered interventions and assessment instruments for PC in this specific patient group.

As a major limitation, we only interviewed patients from a single university medical center in Germany. We also cannot rule out self-selection bias, i.e., the patients who participated may have differed from those who decided not to participate, as neither had we assessed the characteristics of non-participants and drop-outs, nor their reasons for refusing to participate. Our sample’s heterogeneity in terms of age, sex, and diagnoses suggests that the purposeful sampling was successful and our patients proved to differ in their views on and experiences with PC. Yet, the high number of chronic diseases suggests that the patients included in our study might have been frailer. As a further limitation, the questions in our interview guide had not been pilot-tested on patients beforehand. Instead, they were discussed and refined by a committee of experts at the Center for Geriatric Medicine and Gerontology at the University of Freiburg. Finally, a potential researcher bias must be reflected. As our study is aimed at exploring the transferability of the PC model to a specific target population, there might have been a bias in assessing data and over-interpreting the results in a way that supports the model. In order to minimize this risk, team members who worked on the development of the former integrative model of PC were not involved, neither in conducting the focus group interviews, nor in interpreting the results. Furthermore, we used open interview questions and defined explicit coding rules that were applied during data analysis.

## Conclusion

The preferences and expectations of older patients with multimorbidity on patient-centered care are largely covered by the integrative PC model’s dimensions [35]. To establish a population-specific adaptation of the model, we complemented the existing dimensions, adding the new ‘individual care needs related to aging and chronic disease’ dimension. This newly adapted model may allow us to identify any gaps, e.g., regarding the availability of instruments assessing PC, and stimulate the development of new patient-centered interventions and measurement tools.

## Supplementary Information

**Additional file 1.** Diagnoses of participants (assigned to ICD-10 chapters). Tabulated overview of diagnoses within the sample according to ICD-10.

**Additional file 2.** Consolidated criteria for reporting qualitative studies (COREQ): 32-item checklist. Completed consolidated criteria for reporting qualitative studies (COREQ) checklist.

**Additional file 3.** Participant questionnaire. Short questionnaire on socio-demographic and illness-related characteristics.

**Additional file 4.** Focus groups interview guide. Questions of the focus groups interview guide.

## Data Availability

The datasets generated and/or analysed during the current study are not publicly available due the maintaince of confidentiality of our participants and declarations within the written information which participants had agreed on.

## Abbreviations

COREQ: Consolidated criteria for reporting qualitative studies
DEGAM: German College of General Practitioners and Family Physicians
DFG: German Research Foundation
GP: General Practitioner
ICD: International Statistical Classification of Diseases and Related Health Problems
M: Mean
PC: Patient-centeredness
PROSPERO: International Prospective Register of Systematic Reviews
SD: Standard Deviation
SEVERA: Section of Health Care Research and Rehabilitation Research
ZGGF: Centre for Geriatric Medicine and Gerontology
