# Supplementary material for: Patient-centeredness in the multimorbid elderly: a focus group study
Source: BMC Geriatr. 2021 Oct 18;21:567. doi: 10.1186/s12877-021-02448-8 (PMC8522160; doi:10.1186/s12877-021-02448-8)
Supplement: Supplementary file 1 — Additional file 1. Diagnoses of participants (assigned to ICD-10 chapters). Tabulated overview of diagnoses within the sample according to ICD-10. [file 12877_2021_2448_MOESM1_ESM.pdf]

Additional file 1. Diagnoses of participants (assigned to ICD-10 chapters)

ICD chapter	N (%)	1st	2nd	3rd	4th
**I: Certain infectious and parasitic diseases (A00-B99)**	2 (10%)	0	0	1	1
**II: Neoplasms (C00-D48)**	6 (30%)	3	2	0	1
Malignant neoplasms	5	3	1	0	1
In situ neoplasms	1	0	1	0	0
**IV: Endocrine, nutritional and metabolic diseases (E00-E90)**	5 (25%)	2	1	0	2
Diabetes mellitus	3	2	1	0	0
Metabolic disorders	1	0	0	0	1
Disorders of thyroid gland	1	0	0	0	1
**V: Mental and behavioural disorders(F00-F99)**	7 (35%)	0	2	2	3
Affective disorder	5	0	1	2	2
Neurotic, stress-related and somatoform disorders	1	0	1	0	0
Organic, including symptomatic, mental disorders	1	0	0	0	1
**VI: Diseases of the nervous system (G00-G99)**	8 (40%)	3	2	3	0
Polyneuropathy	3	2	1	0	0
Episodic and paroxysmal disorder	3	1	0	2	0
Extrapyramidal and movement disorder	2	0	1	1	0
**VII: Diseases of the eye and adnexa (H00-H59)**	3 (15%)	0	3	0	0
Disorders of lens	2	0	2	0	0
Disorders of choroid and retina	1	0	1	0	0
**VIII: Diseases of the ear and mastoid process (H60-H95)**	4 (20%)	1	1	1	1
Diseases of inner ear	2	0	1	1	0
Other disorders of ear	2	1	0	0	1
**IX: Diseases of the circulatory system (I00-I99)**	19 (95%)	4	4	5	6
Hypertensive diseases	8	3	3	1	1
Ischaemic heart diseases	3	1	0	1	1
Cerebrovascular diseases	4	0	1	1	2
Other forms of heart disease	2	0	0	1	1
Diseases of arteries, arterioles and capillaries	1	0	0	1	0
Diseases of veins, lymphatic vessels and lymph nodes	1	0	0	0	1
**X: Diseases of the respiratory system (J00-J99)**	1 (5%)	1	0	0	0
**XI: Diseases of the digestive system (K00-K93)**	2 (10%)	1	1	0	0
**XIII: Diseases of the musculoskeletal system and connective tissue (M00-M99)**	12 (60%)	5	2	4	1
Arthropathies	3	2	1	0	0
Osteopathies and chondropathies	3	1	0	1	1
Dorsalgia	5	2	1	2	0
Soft tissue disorders	1	0	0	1	0
**XIV: Diseases of the genitourinary system (N00-N99)**	4 (20%)	0	2	1	1
Renal failure	2	0	1	0	1
Diseases of male genital organs	2	0	1	1	0
**XVIII: Symptoms, signs and abnormal clinical and laboratory findings, not elsewhere classified (R00-R99)**	3 (15%)	0	0	1	2
General symptoms and signs	1	0	0	1	0
Abnormal findings on diagnostic imaging and in function studies	1	0	0	0	1
Symptoms and signs involving the circulatory and respiratory systems	1	0	0	0	1
