# Supplementary material for: Patient-centeredness in the multimorbid elderly: a focus group study
Source: BMC Geriatr. 2021 Oct 18;21:567. doi: 10.1186/s12877-021-02448-8 (PMC8522160; doi:10.1186/s12877-021-02448-8)
Supplement: Supplementary file 2 — Additional file 2. Consolidated criteria for reporting qualitative studies (COREQ): 32-item checklist. Completed consolidated criteria for reporting qualitative studies (COREQ) checklist. [file 12877_2021_2448_MOESM2_ESM.pdf]

Additional file 2. Consolidated criteria for reporting qualitative studies (COREQ): 32-item checklist[1]

No	Item	Guide question/description	Response
**DOMAIN 1: RESEARCH TEAM AND REFLEXIVITY**			
**Personal Characteristics**			
1.	Interviewer/facilitator	Which author/s conducted the interview or focus group?	Methods, “data collection”, paragraph 1
2.	Credentials	What were the researcher’s credentials? *E.g. PhD, MD*	Methods, “data collection”, paragraph 1
3.	Occupation	What was their occupation at the time of the study?	Methods, “data collection”, paragraph 1
4.	Gender	Was the researcher male or female?	Methods, “data collection”, paragraph 1
5.	Experience and training	What experience or training did the researcher have?	Methods, “data collection”, paragraph 1
**Relationship with participants**			
6.	Relationship established	Was a relationship established prior to study commencement?	Methods, “data collection”, paragraph 1
7.	Participant knowledge of the interviewer	What did participants know about the researcher? E*.g. personal goals, reasons for doing the research*	Methods, “data collection”, paragraph 1
8.	Interviewer characteristics	What characteristics were reported about the interviewer/facilitator? *E.g. Bias, assumptions, reasons and interests in the research topic*	Methods, “data collection”, paragraph 1
**DOMAIN 2: STUDY DESIGN**			
**Theoretical framework**			
9.	Methodological orientation and Theory	What methodological orientation was stated to underpin the study? *E.g. grounded theory, discourse analysis, ethnography, phenomenology, content analysis*	Methods, “data analysis”, paragraph 1
**Participant selection**			
10.	Sampling	How were participants selected? *E.g. purposive, convenience, consecutive, snowball*	Methods, “participants”, paragraph 1
11.	Method of approach	How were participants approached? *E.g. face-to-face, telephone, mail, email*	Methods, “participants”, paragraph 1
12.	Sample size	How many participants were in the study?	Methods, “participants”, paragraph 1
13.	Non-participation	How many people refused to participate or dropped out? Reasons?	Methods, “participants”, paragraph 1;<br>Discussion, “strengths and limitations”, paragraph 2
**Setting**			
14.	Setting of data collection	Where was the data collected? *E.g. home, clinic, workplace*	Methods, “data collection”, paragraph 1

[1] Developed from: Tong A, Sainsbury P, Craig J. Consolidated criteria for reporting qualitative research (COREQ): a 32-item checklist for interviews and focus groups. International Journal for Quality in Health Care. 2007. Volume 19, Number 6: pp. 349 – 357

15.	Presence of non-participants	Was anyone else present besides the participants and researchers?	Methods, "data collection", paragraph 1
16.	Description of sample	What are the important characteristics of the sample? *E.g. demographic data, date*	Methods, "participants", paragraph 1 and Table 1
**Data collection**			
17.	Interview guide	Were questions, prompts, guides provided by the authors? Was it pilot tested?	Methods, "interview guide, paragraph 1; Discussion, "strengths and limitations", paragraph 2
18.	Repeat interviews	Were repeat interviews carried out? If yes, how many?	N/A
19.	Audio/visual recording	Did the research use audio or visual recording to collect the data?	Methods, "data collection", paragraph 1
20.	Field notes	Were field notes made during and /or after the interview or focus group?	Methods, "data collection", paragraph 1
21.	Duration	What was the duration of the interviews or focus group?	Methods, "data collection", paragraph 1
22.	Data saturation	Was data saturation discussed?	Discussion, "strengths and limitations", paragraph 2
23.	Transcripts returned	Were transcripts returned to participants for comment and/or correction?	N/A
**DOMAIN 3: ANALYSIS AND FINDINGS**			
**Data analysis**			
24.	Number of data coders	How many data coders coded the data?	Methods, "data analysis", paragraph 1
25.	Description of the coding tree	Did authors provide a description of the coding tree?	Table 3
26.	Derivation of themes	Were themes identified in advance or derived from the data?	Methods, "data analysis", paragraph 1
27.	Software	What software, if applicable, was used to manage the data?	Methods, "data analysis", paragraph 1
28.	Participant checking	Did participants provide feedback on the data?	N/A
**Reporting**			
29.	Quotations presented	Were participant quotations presented to illustrate the themes/findings? Was each quotation identified? *E.g. participant number*	Results, paragraph 2-20
30.	Data and findings consistent	Was there consistency between the data presented and the findings?	Discussion, paragraph 2
31.	Clarity of major themes	Were major themes clearly presented in the findings?	Results, paragraph 2-20; Discussion, paragraph 2
32.	Clarity of minor themes	Is there a description of diverse cases or discussion of minor themes?	Results, paragraph 2-20; Tables, Table 3
