# Supplementary material for: Patient-centeredness in the multimorbid elderly: a focus group study
Source: BMC Geriatr. 2021 Oct 18;21:567. doi: 10.1186/s12877-021-02448-8 (PMC8522160; doi:10.1186/s12877-021-02448-8)
Supplement: Supplementary file 3 — Additional file 3. Participant questionnaire. Short questionnaire on socio-demographic and illness-related characteristics. [file 12877_2021_2448_MOESM3_ESM.pdf]

Additional file 3. Participant questionnaire

PATIENT QUESTIONNAIRE

First of all, please state your name. Your name is only needed to assign you a number. It will be removed from the questionnaire later on so that it can no longer be connected with you personally.
Your name: ______________________________

ID (Pseudonym)<br>(to be entered by the researchers)	____________________

In the following section, you will find some questions about yourself and your illness. Please mark the answer that applies to you.				
How old are you?				1
_______ years				
Your gender?				2
male	☐ 1	female	☐ 2	
Your nationality?				3
German	☐ 1	other	☐ 2	
Do you live with a spouse or permanent partner?				4
yes	☐ 1	no	☐ 2	

What is the highest level of education or degree you have obtained?<br>(If you have several degrees, please mark only the <u>highest</u> qualification).	5
No school-leaving certificate	☐ 1
Secondary general school certificate (*Volksschul- oder Hauptschulabschluss*)	☐ 2
General certificate of secondary education (*Mittlere Reife / Realschulabschluss*)	☐ 3
Poly-technical high school (*Polytechnische Oberschule*)	☐ 4
Advanced technical college entrance qualification (*Fachhochschulreife / Abschluss einer Fachoberschule)*)	☐ 5
Qualification for university entrance (*Abitur / Hochschulreife*)	☐ 6
Other school-leaving certificate	☐ 7

The following questions refer to your professional career.					6
Are you still working? (including voluntary work)	yes	☐ 1	no	☐ 2	
Did you retire early?	yes	☐ 1	no	☐ 2	
If so, at the age of: ___________					

Please name the illness or health problem that is currently affecting you the most (the problem can be either physical or psychological)	7
______________________________	
______________________________	

How long have you had this illness?

___	days	8
___	weeks	9
___	months	10
___	years	11

How burdened do you feel by your illness in your everyday life?<br>(Please circle the number that applies to you.)	12

**Not at all**							**Very much**
0	1	2	3	4	5	6	7

THANK YOU VERY MUCH FOR YOUR COOPERATION!
