# Supplementary material for: Patient-centeredness in the multimorbid elderly: a focus group study
Source: BMC Geriatr. 2021 Oct 18;21:567. doi: 10.1186/s12877-021-02448-8 (PMC8522160; doi:10.1186/s12877-021-02448-8)
Supplement: Supplementary file 4 — Additional file 4. Focus groups interview guide. Questions of the focus groups interview guide. [file 12877_2021_2448_MOESM4_ESM.pdf]

Additional file 4. Focus groups interview guide

Research Project
"Availability and Quality of Assessment Instruments on Patient-Centeredness in the Multimorbid Elderly"

FOCUS GROUPS WITH PATIENTS

Interview Guide

1) Introduction to the topic	1 min
There is a demand nowadays to make providing health care as patient-centered as possible. This means that treatment should be adapted to the individual patient's needs, and that the patient be the focus of attention.	

2) Lay concept — 10 min

- **What spontaneously comes to mind when you think of the term patient-centeredness?**
  - In your opinion, what exactly would health care look like if it were more closely centered to the individual patient?
  - What experiences have you had personally with patient-centeredness?
  - What else is important to customize treatment to each patient?

3) Specifities of older patients — 10 min

- **If you think of patient-centered health care, what might be different for older as compared to younger patients?**
  - Do you think older people might have other expectations and needs regarding their treatment than younger people? Or maybe other concerns or fears?
  - Which other aspects should be specifically considered in the treatment of older people?
  - What else do you suppose might be more important to older than to younger patients?

4) Specifities arising from multimorbidity	7-8 min

- **From your perspective, what should be considered in the health care of older patients who suffer from multiple chronic diseases?**
  - Do you believe older people might have different health care expectations and needs when they suffer from multiple chronic diseases?
  - Is there anything else that should be considered if an older patient suffers from several chronic diseases?
  - What should health care providers (e.g. general practitioner, medical specialist, nursing staff) consider especially when dealing with a multimorbid patient?

5) View of relatives	7-8 min

- **What might your relatives say about what needs to be specifically considered in the health care of older patients who suffer from multiple chronic diseases?**
  - What factors do your relatives regard as important to adapt health care better to the individual patient?
  - If we asked your relatives (e.g. your spouse, your children or other related persons) what patient-centered health care meant to them – how would they answer?

6) Questions that dig deeper (optional)	7-8 min

Systemic Variables:

- The therapy of older patients often requires the participation of an entire network of professional caregivers, such as general practitioners, specialists and consultants, clinic staff, health insurance providers, aftercare or rehabilitation providers, as well as relatives. Keeping such a varied group of participants in mind, what do you think is important to achieve patient-centered health care?

For patients being treated:

- Think about your relationship to your physician or to other health care providers such as nursing staff or physiotherapists – what is perhaps important here?
- Leaving medical expertise out of the picture for the moment: What must the therapy-providing team pay special attention to so that you feel well looked after?

Empowerment and Autonomy:

- What do you think older patients would like to contribute to make their treatment more effective?
- What do older patients perhaps prefer to do themselves during their therapy? How might therapy providers support them in that endeavor?

7) Final question	5-10 min

- **If you look back at our interview is there anything you think was missing when discussing the topic of patient-centeredness?**
